# Sparsentan Versus Irbesartan in Pediatric Patients With FSGS

**DOI:** 10.1016/j.ekir.2026.106566

**Published:** 2026-04-22

**Authors:** Michelle N. Rheault, Katherine M. Dell, Kenneth V. Lieberman, Kevin Meyers, Ana Paredes, Edward Murphy, Jula K. Inrig, Radko Komers, Howard Trachtman

**Affiliations:** 1 Division of Pediatric Nephrology, University of Minnesota Medical School, Minneapolis, Minnesota, USA; 2 Center for Pediatric Nephrology and Hypertension, Cleveland Clinic Children’s and Case Western Reserve University, Cleveland, Ohio, USA; 3 Department of Pediatric Nephrology, Hackensack University Medical Center, Hackensack, New Jersey, USA; 4 Division of Nephrology, Department of Pediatrics, Children’s Hospital of Philadelphia, Philadelphia, USA; 5 Niklaus Children’s Hospital, Miami, Florida, USA; 6 Travere Therapeutics, Inc., San Diego, California, USA; 7 Division of Nephrology, Department of Pediatrics, University of Michigan, Ann Arbor, Michigan, USA

**Keywords:** clinical trial, focal segmental glomerulosclerosis, pediatric, proteinuria, sparsentan, therapy

## Abstract

**Introduction:**

Sparsentan, a nonimmunosuppressive dual endothelin angiotensin receptor antagonist, led to rapid and sustained proteinuria reductions versus maximum labeled dose irbesartan in patients with focal segmental glomerulosclerosis (FSGS) in the phase 3 DUPLEX trial (NCT03493685). Here, we describe the efficacy and safety of sparsentan in the pediatric patients enrolled in DUPLEX.

**Methods:**

DUPLEX was a 108-week randomized trial of sparsentan versus irbesartan (active control) in patients with FSGS (excluding known secondary causes). A *post hoc* analysis assessed efficacy and safety in the pediatric subgroup (8 to < 18 years). Outcomes included proteinuria (urine protein-to-creatinine ratio [UPCR]) changes from baseline; rates of complete remission (CR) of proteinuria (UPCR of < 0.3 g/g); FSGS partial remission end point (UPCR of ≤ 1.5 g/g and > 40% reduction from baseline); UPCR of < 0.5, < 0.7, or < 1.0 g/g at any time; rates of kidney failure; and safety.

**Results:**

Thirty-five pediatric patients received sparsentan (*n* = 16) or irbesartan (*n* = 19). UPCR reductions were observed as early as week 6 and sustained through 108 weeks (mean reduction at 108 weeks: sparsentan, 39.5% vs. irbesartan, 24.9%). More patients in the sparsentan arm than the irbesartan arm achieved low proteinuria thresholds, including CR (12.5% vs. 5.3%) and the FSGS partial remission end point (56.3% vs. 36.8%). Sparsentan and irbesartan had similar safety profiles, with comparable incidence and severity of adverse events.

**Conclusion:**

In this *post hoc* analysis of pediatric patients with FSGS, sparsentan was generally well tolerated and clinically meaningful reductions in proteinuria were observed, consistent with the overall DUPLEX population.

## Graphical abstract

**Figure:**
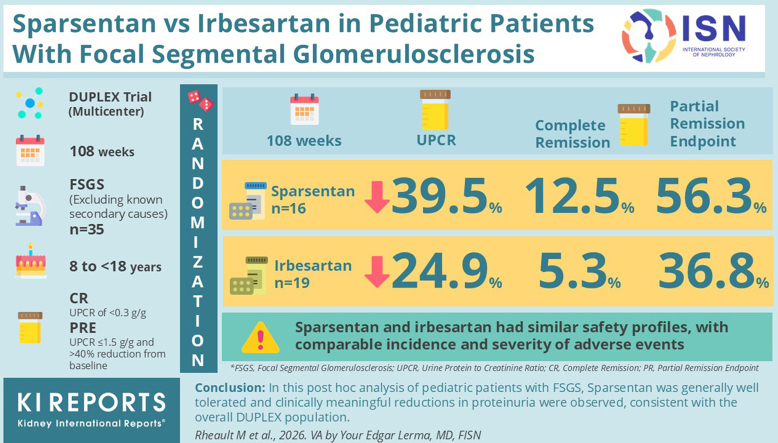

FSGS is a rare, progressive kidney condition characterized by a histologic pattern of podocyte and glomerular injury.^1^^,^^2^ It is among the leading causes of nephrotic syndrome and kidney failure in children globally.^3^, ^4^, ^5^, ^6^ Moreover, approximately 30% to 60% of all patients with FSGS progress to kidney failure or transplant within 10 years of diagnosis.^7^^,^^8^ There are currently no US Food and Drug Administration–approved therapies for FSGS, and approximately half of children with the condition do not respond to the current standard of care.^3^^,^^9^ As such, there is an urgent need for safe and effective treatments for those with FSGS, including pediatric patients.^10^

In FSGS, proteinuria is both a consequence of podocyte injury and a risk factor for further kidney damage, and elevated proteinuria is associated with an increased risk of progression to kidney failure.^2^^,^^11^^,^^12^ Conversely, proteinuria reduction across a range of UPCR thresholds and response definitions is a clinically meaningful outcome associated with a reduced risk of kidney failure,^2^^,^^12^, ^13^, ^14^ supporting lowering proteinuria as a key treatment goal for patients with FSGS.

The endothelin-1 and angiotensin II (Ang II) signaling pathways are key mediators of kidney damage that are upregulated in FSGS and act in tandem via the endothelin type A and Ang II type 1 receptors to exacerbate podocyte injury, leading to proteinuria, inflammation, and fibrosis.^15^, ^16^, ^17^, ^18^ Sparsentan is a nonimmunosuppressive dual endothelin angiotensin receptor antagonist that targets kidney damage by simultaneously blocking both the endothelin-1 and Ang II signaling pathways.^15^^,^^19^ Sparsentan is approved in the US and Europe for the treatment of adults with IgA nephropathy based on superior proteinuria reduction and kidney function preservation compared with maximum labeled dose irbesartan observed in the phase 3 PROTECT trial.^20^^,^^21^ In the phase 3 DUPLEX trial, patients with FSGS treated with sparsentan had rapid and sustained reductions in proteinuria compared with those who received maximum labeled dose irbesartan; these reductions occurred within 6 weeks and were sustained through 108 weeks.^22^ This *post hoc* analysis evaluated the efficacy and safety of sparsentan in pediatric patients, a planned subgroup of interest in DUPLEX.

## Methods

### Study Design

The design of the phase 3, double-blind DUPLEX trial (NCT03493685; registration date, April 3, 2018) has been previously described.^22^^,^^23^ In brief, patients aged 8 to 75 years with biopsy-proven FSGS or a documented genetic cause of FSGS (patients with known secondary causes of FSGS were excluded), a UPCR ≥ 1.5 g/g, and an estimated glomerular filtration rate (eGFR) of ≥ 30 ml/min per 1.73 m^2^ were eligible for enrollment. Patients were randomly assigned (1:1) to receive sparsentan or irbesartan for 108 weeks, after a 2-week washout period during which previous renin-angiotensin system inhibitors were discontinued. Following 2 weeks of treatment with sparsentan 400 mg/day or irbesartan 150 mg/day, doses were titrated to the target levels of sparsentan 800 mg/day or irbesartan 300 mg/day. Patients weighing ≤ 50 kg received one-half the otherwise specified doses; the dose was increased if the patient’s weight exceeded 50 kg during the trial. This *post hoc* exploratory analysis included pediatric patients aged 8 to < 18 years enrolled in DUPLEX.

### Outcomes

Proteinuria-based outcomes for this subgroup analysis were percentage change from baseline in UPCR and achievement of the following: (i) complete remission of proteinuria (CR; defined as UPCR of < 0.3 g/g), (ii) the FSGS partial remission end point (UPCR of ≤ 1.5 g/g and > 40% reduction from baseline),^24^ and (iii) UPCR thresholds of < 0.5 g/g, < 0.7 g/g, or < 1.0 g/g at any time during the 108-week double-blind treatment period. Rates of kidney failure (defined as sustained eGFR of < 15 ml/min per 1.73 m^2^ or renal replacement therapy) and a composite kidney end point (confirmed 40% reduction in eGFR, kidney failure, or death) were assessed through 112 weeks. Blood pressure was categorized as normal, elevated, stage 1 hypertension, or stage 2 hypertension as previously described.^25^ Changes in blood pressure categories from baseline to week 108 were assessed in patients aged < 18 years of age at week 108 (patients who turned 18 during the study were not included [sparsentan *n* = 6; irbesartan *n* = 9]). The proportion of patients who required new initiation or intensification of immunosuppressive therapy (IST) is also reported. Safety outcomes included the incidence and severity of treatment-emergent adverse events (TEAEs).

### Statistical Analyses

Statistical analyses were conducted using Statistical Analysis Systems (version 9.4 or later, SAS Institute Inc., Cary, NC) in accordance with previously described methods.^22^ Demographic and baseline characteristics are summarized using descriptive statistics. UPCR was calculated as the geometric mean of 2 to 3 first morning void urine samples collected within 5 days before each visit. The percentage change in UPCR was analyzed using a mixed model for repeated measures with the natural log (UPCR) as the dependent variable, treatment, baseline UPCR, visit, treatment-by-visit interaction, and randomization stratification factors as fixed effects, patient as a random effect, and an unstructured covariance model. The estimated least squares mean change in UPCR with 95% confidence interval [CI] was back transformed as (exp[LS mean change from baseline in natural log(UPCR)] – 1) × 100. The percentages of patients reaching low proteinuria thresholds (CR, UPCR of < 0.5 g/g, < 0.7 g/g, or < 1.0 g/g, and the FSGS partial remission end point), kidney failure, or the composite kidney end point are reported with 95% CIs generated from an exact binomial distribution. The corresponding relative risks (RRs) with 95% CIs were obtained from a Cochran-Mantel-Haenszel test accounting for randomization stratification factors.

### Ethical Considerations

Patients provided written informed consent or, where applicable, assent (with parent’s and/or legal guardian’s consent) before enrollment. This study was approved by institutional review boards or independent ethics committees at each site and was conducted in accordance with the principles of Good Clinical Practice and the Declaration of Helsinki.

## Results

### Patient Population and Dose

A total of 35 pediatric patients were enrolled in the DUPLEX trial and randomly assigned to receive sparsentan (*n* = 16) or maximum labeled dose irbesartan (*n* = 19). Baseline characteristics were generally comparable between treatment arms (Table 1). Most patients in both treatment arms were adolescents (aged 10 to < 18 years), with median ages of 14.5 and 14.0 years in the sparsentan and irbesartan arms, respectively. On average, baseline proteinuria levels were similar between treatment arms (median UPCR: sparsentan 4.7 g/g, irbesartan 5.2 g/g). A higher percentage of patients in the sparsentan arm (14/16 [88%]) than in the irbesartan arm (9/19 [47%]) received an angiotensin-converting enzyme inhibitor or an Ang II receptor blocker before study entry. Six patients in the sparsentan arm (38%) and 5 in the irbesartan arm (26%) received concurrent IST for kidney indications at baseline. Patients in the sparsentan arm had, on average, lower median eGFR than those in the irbesartan arm (72 ml/min per 1.73 m^2^ vs. 82 ml/min per 1.73 m^2^, respectively).

**Table 1:** Baseline characteristics (*N* = 35)

	Sparsentan *n* = 16	Irbesartan *n* = 19	Total *N* = 35
Age, yrs			
Median (IQR)	14.5 (13.0–16.0)	14.0 (12.0–17.0)	14.0 (12.0–16.0)
Range, minimum-maximum	9–17	9–17	9–17
Male, *n* (%)	5 (31)	7 (37)	12 (34)
Race, *n* (%)^a^			
White	12 (75)	13 (68)	25 (71)
Black or African American	3 (19)	2 (11)	5 (14)
Other	3 (19)	3 (16)	6 (17)
Asian	0	1 (5)	1 (3)
Ethnicity, *n* (%)			
Not Hispanic or Latino	9 (56)	12 (63)	21 (60)
Hispanic or Latino	7 (44)	7 (37)	14 (40)
Weight, median (IQR), kg	55.5 (49.4–66.3)	59.3 (50.3–82.7)	58.2 (49.6–72.5)
eGFR, ml/min per 1.73 m^2^			
Median (IQR)	71.5 (52.5–107.0)	82.0 (59.0–149.0)	79.0 (53.0–124.0)
Range, minimum-maximum	36–172	31–208	31–208
UPCR, median (IQR), g/g	4.7 (3.3–7.3)	5.2 (3.4–7.6)	5.0 (3.3–7.3)
Blood pressure, median (IQR), mm Hg			
Systolic	115 (110–126)	116 (111–129)	116 (111–128)
Diastolic	76 (70–85)	78 (67–84)	78 (68–84)
Blood pressure category, *n* (%)^b^			
Normal blood pressure	8 (50)	8 (42)	16 (46)
Elevated blood pressure	1 (6)	2 (11)	3 (9)
Stage 1 hypertension	5 (31)	6 (32)	11 (31)
Stage 2 hypertension	2 (13)	3 (16)	5 (14)
Pathogenic or likely pathogenic genetic variants, *n* (%)^c^^,^^d^			
*APOL1* high-risk variants	1 (6)	2 (11)	3 (9)
Monogenic podocyte gene variants^e^	6 (38)	2 (11)	8 (23)
Pretreatment ACEi or ARB use, *n* (%)^f^	14 (88)	9 (47)	23 (66)
ACEi	10 (63)	8 (42)	18 (51)
ARB	6 (38)	1 (5)	7 (20)
Baseline use of IST for kidney indications, *n* (%)^g^	6 (38)	5 (26)	11 (31)
Calcineurin inhibitors	6 (38)	4 (21)	10 (29)
Mycophenolate sodium or mofetil	2 (13)	1 (5)	3 (9)
Glucocorticoids	3 (19)	0	3 (9)
Documented history of nephrotic syndrome, *n* (%)^h^			
Yes	11 (68.8)	10 (52.6)	21 (60)
No	5 (31.3)	9 (47.4)	14 (40)

ACEi, angiotensin-converting enzyme inhibitor; ARB, angiotensin II receptor blocker; *APOL1*, apolipoprotein L1; *CD2AP,* CD2-associated protein; *COL4A3-5,* collagen type IV (COL4) α3, α4, and α5; eGFR, estimated glomerular filtration rate; IQR, interquartile range; *INF2*, inverted formin 2; IST, immunosuppressive therapy; *LMX1B*, LIM homeobox transcription factor 1β; *NPHS1*, nephrin; *NPHS2*, podocin; *TRPC6*, transient receptor potential cation channel subfamily C member 6; UPCR, urine protein-to-creatinine ratio; *WT1*, WT1 transcription factor.

a Patients may have identified as more than 1 race.

b For patients aged ≥ 13 years: Normal blood pressure was defined as systolic blood pressure < 120 mm Hg and diastolic blood pressure < 80 mm Hg; elevated blood pressure as systolic blood pressure 120–129 mm Hg and diastolic blood pressure < 80 mm Hg; stage 1 hypertension as systolic blood pressure 130–139 mm Hg or diastolic blood pressure 80–89 mm Hg; and stage 2 hypertension as systolic blood pressure ≥ 140 mm Hg or diastolic blood pressure ≥ 90 mm Hg.^25^ For patients aged < 13 years: Normal blood pressure was defined as < 90th percentile; elevated blood pressure as ≥ 90th to < 95th percentile or systolic/diastolic blood pressure of ≥ 120/80 mm Hg to < 95th percentile (whichever is lower); stage 1 hypertension as ≥ 95th to < 95th percentile + 12 mm Hg or systolic/diastolic blood pressure of 130/80 to 139/89 mm Hg (whichever is lower); and stage 2 hypertension as ≥ 95th percentile + 12 mm Hg or systolic/diastolic blood pressure of ≥140/90 mm Hg (whichever is lower).^25^

c Genetic variants were evaluated in post hoc analyses after completion of the double-blind period.

d None of the patients had identified *COL4A3-5* variants.

e Includes *NPHS2, CD2AP, INF2, LMX1B, NPHS1, TRPC6*, and *WT1* variants.

f Patients may have had previous treatment with both an ACEi and an ARB before the initiation of study medication.

g Medications initiated before and continued after first dose of study drug.

h Patients were considered to have documented history of nephrotic syndrome if the term “nephrotic syndrome” was present in their medical history or if all of the following conditions were met at any visit before the first dose of study drug: UPCR > 2.0 g/g, serum albumin level < 3.0 g/l, and abnormal edema observed on physical examination.

In total, 11 pediatric patients had identified genetic causes of FSGS attributed to monogenic podocyte gene variants or apolipoprotein L1 high-risk genotypes; none had identified collagen type IV alpha 3 to 5 chain variants (Table 1). Genetic causes of FSGS were more common in the sparsentan arm (*n* = 6 with monogenic podocyte gene variants; *n* = 1 with an apolipoprotein L1 high-risk genotype) compared with the irbesartan arm (*n* = 2 with monogenic podocyte gene variants; *n* = 2 with apolipoprotein L1 high-risk genotypes). Outcomes in patients with genetic FSGS from DUPLEX have been reported elsewhere.^26^

**Figure 1:**
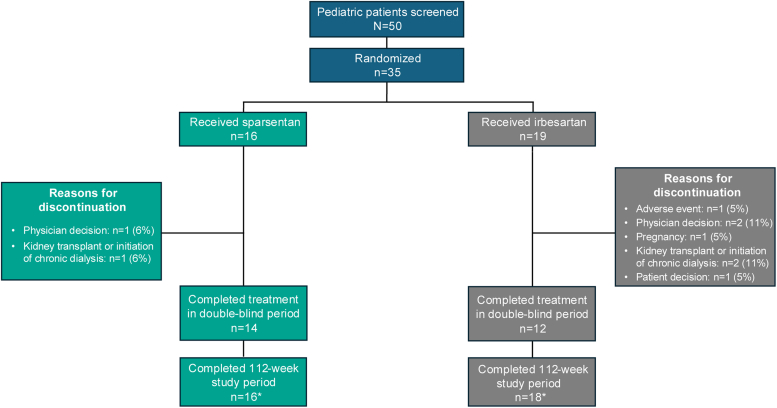
Patient disposition. ∗Includes patients who discontinued treatment but completed visits through the planned study duration of 112 weeks.

Sixteen patients (100%) in the sparsentan arm and 18 (95%) in the irbesartan arm completed the planned study duration of 112 weeks; 14 (88%) and 12 (63%), respectively, completed double-blind treatment. Reasons for treatment discontinuation are summarized in Figure 1.

In total, 94% of pediatric patients (15/16) in the sparsentan arm and 95% of patients (18/19) in the irbesartan arm received the target doses. The median (minimum-maximum) weight-based doses over the 108-week treatment period were 8.8 mg/kg (3.3–13.5) of sparsentan and 3.5 mg/kg (2.4–5.5) of irbesartan.

### Efficacy

#### Proteinuria

**Figure 2:**
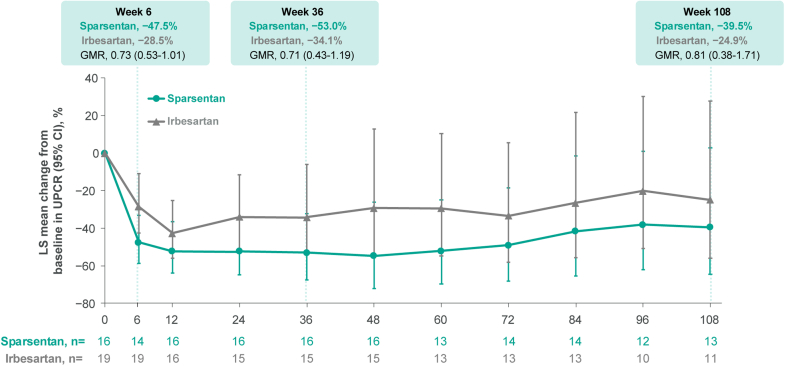
Change in UPCR from baseline∗. GMR, geometric mean ratio; LS, least squares; UPCR, urine protein-to-creatinine ratio. ∗Sample size values reflect the number of evaluable patients who had at least 2 first morning void urine samples at each visit.

As early as 6 weeks, patients receiving sparsentan demonstrated a numerically larger reduction in proteinuria from baseline compared with those receiving irbesartan (Figure 2), with a geometric least square mean reduction of 47.5% (95% CI: 33.2–58.8) in the sparsentan arm and 28.5% (95% CI: 11.0–42.5) in the irbesartan arm (geometric mean ratio of percent reduction: 0.73; 95% CI: 0.53–1.01). This reduction was sustained throughout the treatment period, with a geometric least square mean reduction at 108 weeks of 39.5% (95% CI: 2.8–64.4) in the sparsentan arm and 24.9% (95% CI: 27.8–55.9) in the irbesartan arm (geometric mean ratio of percent reduction, 0.81; 95% CI: 0.38–1.71).

**Figure 3:**
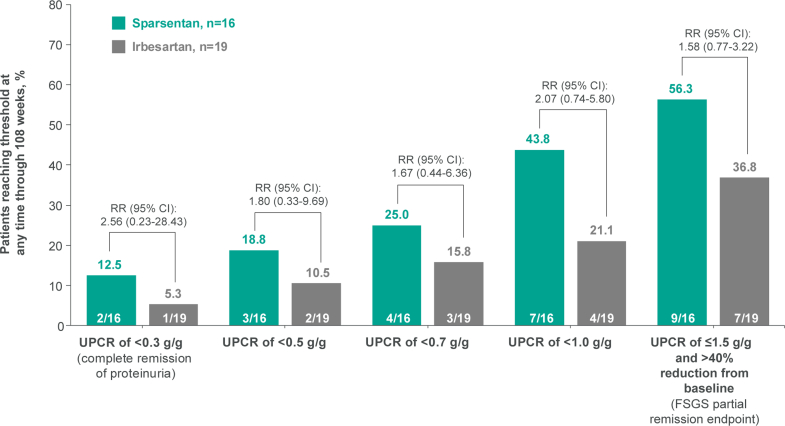
Patients achieving low proteinuria thresholds with sparsentan versus irbesartan. FSGS, focal segmental glomerulosclerosis; RR, relative risk; UPCR, urine protein-to-creatinine ratio.

Compared with the irbesartan arm, a numerically higher proportion of pediatric patients in the sparsentan arm achieved CR at any time during the 108-week double-blind trial period (Figure 3), with 12.5% (2/16) in the sparsentan arm versus 5.3% (1/19) in the irbesartan arm reaching CR (RR: 2.56; 95% CI: 0.23–28.43). Consistent trends were observed for proteinuria reductions to UPCR of < 0.5 g/g, < 0.7 g/g, and < 1.0 g/g and the FSGS partial remission end point (Figure 3), with 18.8% (3/16) in the sparsentan arm versus 10.5% (2/19) in the irbesartan arm achieving UPCR of < 0.5 g/g (RR: 1.80; 95% CI: 0.33–9.69), 25.0% (4/16) versus 15.8% (3/19) achieving UPCR of < 0.7 g/g (RR: 1.67; 95% CI: 0.44–6.36), 43.8% (7/16) versus 21.1% (4/19) achieving UPCR of < 1.0 g/g (RR: 2.07; 95% CI: 0.74–5.80), and 56.3% (9/16) versus 36.8% (7/19) achieving the FSGS partial remission end point (RR: 1.58; 95% CI: 0.77–3.22) at any time.

#### Kidney Failure

**Figure 4:**
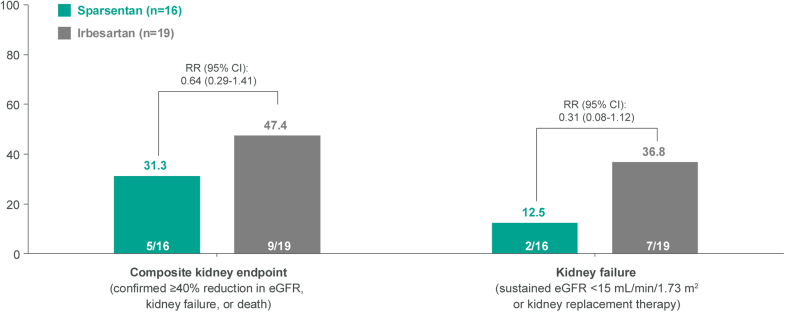
Proportion of patients who progressed to kidney failure end points. eGFR, estimated glomerular filtration rate; RR, relative risk.

Fewer patients in the sparsentan arm progressed to the composite kidney end point or kidney failure during the study compared with those in the irbesartan arm (Figure 4). A total of 5 patients (31.3%) in the sparsentan arm and 9 (47.4%) in the irbesartan arm reached the composite kidney end point (RR: 0.64; 95% CI: 0.29–1.41), and a total of 2 patients (12.5%) in the sparsentan arm and 7 (36.8%) in the irbesartan arm had kidney failure (RR: 0.31; 95% CI: 0.08–1.12) during the trial.

#### Changes in Blood Pressure and IST Use

At baseline, similar proportions of patients had stage 1 or 2 hypertension in the sparsentan and irbesartan arms (in total, 44% [7/16] and 47% [9/19], respectively). Changes from baseline to week 108 were assessed in patients who were < 18 years of age at week 108. Among patients with normal blood pressure at baseline, most (4/6 in the sparsentan arm and 4/5 in the irbesartan arm) had normal blood pressure at week 108, whereas 2 in the sparsentan arm and 1 in the irbesartan arm had elevated blood pressure or stage 1 or 2 hypertension at week 108 (Supplementary Table S1). Among those with stage 1 or 2 hypertension at baseline, blood pressure was within the normal range in 2 of 4 patients in the sparsentan arm and all patients (3/3) in the irbesartan arm at week 108.

Although a numerically higher proportion of patients in the sparsentan arm were receiving concurrent IST for kidney indications at baseline (Table 1), fewer patients in the sparsentan arm (6% [1/16]) than in the irbesartan arm (26% [5/19]) required initiation of new or intensification of ongoing IST during the double-blind period.

### Safety

TEAEs occurred in 94% of pediatric patients (15/16) in the sparsentan arm and in all patients in the irbesartan arm (Table 2). The most common TEAEs included COVID-19, dizziness, diarrhea, pyrexia, increased blood creatinine, and anemia. Serious TEAEs (SAEs) occurred in 8 patients (50%) in the sparsentan arm and 12 (63%) in the irbesartan arm. The most common SAEs included chronic kidney disease, COVID-19, and acute kidney injury. There were no TEAEs of acute kidney injury in the sparsentan arm. There were no TEAEs leading to treatment discontinuation in the sparsentan arm and 2 in the irbesartan arm. No TEAEs led to death in either arm.

**Table 2:** Treatment-emergent adverse events

Patients with TEAEs, *n* (%)^a^	Sparsentan *n* = 16	Irbesartan *n* = 19	Total *N* = 35
Any TEAEs	15 (94)	19 (100)	34 (97)
SAEs	8 (50)	12 (63)	20 (57)
TEAEs leading to treatment discontinuation	0	2 (11)	2 (6)
TEAEs leading to death	0	0	0
TEAEs of interest			
Hyperkalemia	2 (13)	2 (11)	4 (11)
Hypotension	3 (19)	1 (5)	4 (11)
Abnormal liver function test^b^	0	0	0
Most common TEAEs (> 20% of patients)			
COVID-19	6 (38)	5 (26)	11 (31)
Dizziness	5 (31)	3 (16)	8 (23)
Diarrhea	4 (25)	3 (16)	7 (20)
Pyrexia	4 (25)	1 (5)	5 (14)
Blood creatinine increased	4 (25)	0	4 (11)
Anemia	4 (25)	3 (16)	7 (20)
Chronic kidney disease	2 (13)	7 (37)	9 (26)
Acute kidney injury^c^	0	4 (21)	4 (11)
Most common SAEs (> 20% of patients)			
Chronic kidney disease	2 (13)	7 (37)	9 (26)
COVID-19	4 (25)	4 (21)	8 (23)
Acute kidney injury	0	4 (21)	4 (11)

ALT, alanine aminotransferase; AST, aspartate aminotransferase; SAE, serious treatment-emergent adverse event; TEAE, treatment-emergent adverse event; ULN, upper limit of normal.

a All adverse events were coded per the Medical Dictionary for Regulatory Activities version 23.0.

b New elevation in ALT or AST > 3× ULN (with or without elevation of total serum bilirubin to > 2× ULN) or 2× increase from baseline in ALT or AST in a patient who had elevated values before starting study medication.

c Increased serum creatinine of ≥ 0.3 mg/dl within 48 hours, increased serum creatinine of ≥ 1.5× baseline that is known or presumed to have occurred within the previous 7 days, or urine volume < 0.5 ml/kg/hour for ≥ 6 hours.

TEAEs of hyperkalemia occurred in 2 patients each in the sparsentan (13%) and irbesartan (11%) arms, and TEAEs of hypotension occurred in 3 patients (19%) in the sparsentan arm and in 1 patient (5%) in the irbesartan arm; none of these events were serious. There were no pediatric patients with abnormal liver function tests—defined as new elevations in alanine aminotransferase or aspartate aminotransferase to > 3× the upper limit of normal (with or without elevation of total serum bilirubin to > 2× upper limit of normal) or 2× increase from baseline in alanine aminotransferase or aspartate aminotransferase in a patient who had elevated values before starting study medication—in either arm.

## Discussion

Pediatric patients with FSGS experience high morbidity compared with adult patients despite the current standard of care, with 1 study reporting that 37% of those with biopsy-proven FSGS progress to kidney failure or death within 10 years of diagnosis.^7^ In line with this, over one-third of pediatric patients who received maximum labeled dose of irbesartan in the active control arm experienced kidney failure over approximately 2 years in DUPLEX, underscoring the unmet need for well-tolerated and effective treatments for this patient population.

Consistent with findings from the overall DUPLEX population,^22^^,^^27^ rapid and sustained reductions in UPCR were observed in pediatric patients with FSGS who received sparsentan. Compared with the irbesartan arm, more pronounced proteinuria reductions were observed in the sparsentan arm throughout the treatment period. In addition, more patients in the sparsentan arm achieved low proteinuria levels across a range of UPCR response thresholds compared with those in the irbesartan arm. The observed reductions in proteinuria are thought to be clinically meaningful, as lower proteinuria is associated with slower kidney function decline and a lower risk of kidney failure in patients with FSGS.^7^ Similar to published evidence on complete remission of proteinuria^14^^,^^24^ and the FSGS partial remission end point,^28^ recent large-scale analyses of registry and clinical trial data (PARASOL) that included both adult and pediatric patients demonstrated that achieving low proteinuria, across a range of UPCR thresholds, including but not limited to UPCR of < 0.5 g/g, < 0.7 g/g, and < 1.0 g/g, after 2 years, is associated with meaningful reduction in the long-term risk of progression to kidney failure^13^ and a delay in the need for kidney transplant or dialysis.

In line with the association between low proteinuria and an increased likelihood of preserved kidney function,^13^^,^^14^^,^^29^ fewer pediatric patients in the sparsentan arm reached kidney failure or the composite kidney end point, despite having numerically lower median eGFR at baseline. The lower rate of kidney failure is consistent with observations in the full study population^22^ and may support the nephroprotective benefit of sparsentan in pediatric patients with FSGS. The comparable efficacy of sparsentan in pediatric and adult patients suggests that there are no age-related differences in the involvement of Ang II or endothelin signaling as mediators of kidney injury^15^^,^^18^^,^^19^ in patients with FSGS.

Fewer patients in the sparsentan arm required intensification or initiation of IST following the onset of treatment compared with those in the irbesartan arm. Although we have not performed separate analyses to assess the impact of newly initiated or intensified IST use in this subset of patients, it is possible that the higher frequency of such IST use in the irbesartan arm (26%) compared with the sparsentan arm (6%) may have contributed to the magnitude of observed proteinuria reductions. Further, the inclusion of a 2-week washout period during which patients discontinued pretreatment angiotensin-converting enzyme inhibitor or Ang II receptor blocker was expected to mitigate the impact of such previous treatment. However, we cannot exclude the possibility that higher rate of pretreatment angiotensin-converting enzyme inhibitor or Ang II receptor blocker in the sparsentan arm (88%) compared with the irbesartan arm (47%) may have contributed to the observed reductions in proteinuria in this subgroup of patients.

Of note, 6 pediatric patients (38%) in the sparsentan arm had genetic FSGS attributed to a monogenic podocyte gene variant. Although we have not analyzed outcomes separately for this subgroup of pediatric patients due to the limited number of such patients in the active control irbesartan arm (*n* = 2 [11%]), the meaningful reduction in proteinuria observed with sparsentan in a population that included over one-third of patients with monogenic podocyte gene variants is notable, given that such patients are often resistant to other therapeutic interventions.^2^^,^^30^ Consistent with observations in the pediatric subgroup, in an analysis of all patients (adult and pediatric) with genetic causes of FSGS in DUPLEX, sparsentan led to rapid and sustained reductions in proteinuria compared with irbesartan.^26^ Together, findings support the antiproteinuric benefit of sparsentan in pediatric patients with FSGS, including those with genetic causes.

Sparsentan was well tolerated at doses up to 800 mg/day, with an overall safety profile—including impact on blood pressure, rates of hypotension, and rates of hyperkalemia—similar to that of maximum labeled dose irbesartan; the safety profile in pediatric patients with FSGS was largely consistent with the overall study population^22^ and the broad clinical trial experience.^31^^,^^32^ Although the occurrence of SAEs among pediatric patients (sparsentan, 50%; irbesartan, 63%) was slightly higher than that observed in the overall study population (sparsentan, 37%; irbesartan, 44%),^22^ this may be attributed to a relatively higher proportion of SAEs of COVID-19, which accounted for one-half of SAEs in the sparsentan arm and one-third of SAEs in the irbesartan arm among pediatric patients.

This subgroup analysis has several limitations. The relatively small sample size of pediatric patients and the exploratory nature of the analysis preclude assessment of statistical significance between treatment arms and may constrain the generalizability and precision of results. Additionally, there is a relative underrepresentation of Black or African American patients, as previously noted in the overall study population.^22^ Finally, whereas the rate of eGFR decline was the primary end point in DUPLEX,^22^ large-scale analyses have demonstrated that its high degree of variability limits its feasibility as a clinical trial end point in patients with FSGS.^13^ In particular, a long duration of follow-up and large sample sizes are needed to detect changes in eGFR in this rare patient population reliably.^13^ Relatively large decreases in eGFR, including those resulting from amelioration of glomerular hyperfiltration, also contribute to the challenges in assessment and interpretation of eGFR slope changes in this population.^33^ As such, we have refrained from reporting eGFR outcomes in this subgroup of patients, focusing instead on proteinuria and kidney failure end points.^13^ Despite these limitations, given the paucity of clinical trial data from pediatric patients with FSGS, these analyses nonetheless provide valuable insights into this patient population.

Overall, the results demonstrate that, in the subgroup of pediatric patients with FSGS enrolled in DUPLEX, sparsentan was well tolerated and clinically meaningful reductions in proteinuria were observed, including higher rates of complete remission of proteinuria and the FSGS partial remission end point in the sparsentan arm compared with the irbesartan arm. Further, fewer patients in the sparsentan arm progressed to kidney failure than those in the irbesartan arm. Together, findings support the antiproteinuric and nephroprotective benefit of sparsentan in pediatric patients with FSGS.

## Disclosure

All authors received medical writing assistance and editorial support funded by Travere Therapeutics, Inc. MNR reports ownership interest in Microsoft and Protolabs; consultancy fees from Calliditas Therapeutics and Otsuka Pharmaceutical Co., Ltd.; research funding from Aurinia Pharmaceuticals, Inc., Chinook Therapeutics, Inc., Kaneka Pharma America, LLC, River 3 Renal, and Travere Therapeutics, Inc.; royalties from Wolters Kluwer; and participation on data safety monitoring or advisory boards for the Alport Syndrome Foundation and NephJCl. KMD reports support from NIH R01-DK114425 and the PKD Foundation and data safety monitoring or advisory board participation for AMAG Pharmaceuticals and Vertex Pharmaceuticals. AP is a clinical investigator for the DUPLEX trial which is sponsored by Travere Therapeutics, Inc. EM, JKI, and RK are employees and stockholders of Travere Therapeutics, Inc. HT reports consultancy fees from Aclipse, Akebia, Alentis, Alexion/AstraZeneca, Apellis, Astellas, Biogen, Boehringer Ingelheim, Bristol Meyers Squibb, ChemoCentryx, Dimerix, Eloxx Pharmaceuticals, Kaneka, Kidney International, Maze Therapeutics, Natera (RenaSight), NephCure, OneForBio, Otsuka, PhaseV, ProKidney, Travere Therapeutics, Inc., Vera, Walden; ownership and/or stock in Aclipse and PhaseV; honoraria from Astellas and Reata; advisory board participation for Boehringer Ingelheim, Maze Therapeutics, Otsuka, ProKidney, Travere Therapeutics, Inc., and Vera; data safety monitoring board participation for ANCA vasculitis (ChemoCentryx), bumetanide-seizure, Otsuka, and RIVUR trials; steering committee participation for Dimerix, Travere Therapeutics, Inc., and Vera; and has served on the MEDCAC Committee, board of directors for Kidney Health Initiative, and editorial boards for *Pediatric Nephrology, Kidney360, Glomerular Diseases*, and *Expert Opinion on Emerging Drugs*. KVL and KM have no competing interests.

## Supplementary Material

Supplementary file (PDF) **Table S1.** Change in blood pressure categories from baseline to week 108. CONSORT 2025 Checklist.

## Footnotes

Supplementary file (PDF). **Table S1.** Change in blood pressure categories from baseline to week 108. CONSORT 2025 Checklist.
