# Supplementary material for: Sparsentan Versus Irbesartan in Pediatric Patients With FSGS
Source: Kidney Int Rep. 2026 Apr 22;11(7):106566. doi: 10.1016/j.ekir.2026.106566 (PMC13213750; doi:10.1016/j.ekir.2026.106566)
Supplement: Supplementary file (PDF) — Table S1. Change in blood pressure categories from baseline to week 108. CONSORT 2025 Checklist. [file mmc1.pdf]

SUPPLEMENT TO:

Sparsentan vs Irbesartan in Pediatric Patients With Focal Segmental Glomerulosclerosis

Rheault MR, et al.

CONTENTS

Supplementary Table S1. Change in blood pressure categories from baseline to week 108 1

CONSORT 2025 Checklist 2

Supplementary Table S1. Change in blood pressure categories from baseline to week 108[a]

Baseline BP category, *n* (%)[b]	Week 108 post-baseline BP category, n (%)				
	Normal	Elevated	Stage 1 hypertension	Stage 2 hypertension	Total
**Sparsentan (*n* = 10)[c]**					
Normal	4 (40)	1 (10)	0 (0)	1 (10)	6 (60)
Elevated	0 (0)	0 (0)	0 (0)	0 (0)	0 (0)
Stage 1 hypertension	1 (10)	0 (0)	1 (10)	1 (10)	3 (30)
Stage 2 hypertension	1 (10)	0 (0)	0 (0)	0 (0)	1 (10)
Total	6 (60)	1 (10)	1 (10)	2 (20)	10 (100)
**Irbesartan (*n* = 10)[c]**					
Normal	4 (40)	1 (10)	0 (0)	0 (0)	5 (50)
Elevated	0 (0)	2 (20)	0 (0)	0 (0)	2 (20)
Stage 1 hypertension	3 (30)	0 (0)	0 (0)	0 (0)	3 (30)
Stage 2 hypertension	0 (0)	0 (0)	0 (0)	0 (0)	0 (0)
Total	7 (70)	3 (30)	0 (0)	0 (0)	10 (100)

BP, blood pressure.

[a]For patients aged ≥13 years: Normal blood pressure was defined as systolic blood pressure <120 mm Hg and diastolic blood pressure <80 mm Hg; elevated blood pressure as systolic blood pressure 120-129 mm Hg and diastolic blood pressure <80 mm Hg; stage 1 hypertension as systolic blood pressure 130-139 mm Hg or diastolic blood pressure 80-89 mm Hg; and stage 2 hypertension as systolic blood pressure ≥140 mm Hg or diastolic blood pressure ≥90 mm Hg.[1] For patients aged <13 years: Normal blood pressure was defined as <90th percentile; elevated blood pressure as ≥90th to <95th percentile or systolic/diastolic blood pressure of ≥120/80 mm Hg to <95th percentile (whichever is lower); stage 1 hypertension as ≥95th to <95th percentile + 12 mm Hg or systolic/diastolic blood pressure of 130/80 to 139/89 mm Hg (whichever is lower); and stage 2 hypertension as ≥95th percentile + 12 mm Hg or systolic/diastolic blood pressure of ≥140/90 mm Hg (whichever is lower).[1] [b]Baseline is defined as the last non-missing value on or prior to the start of the dosing. [c]Patients aged ≥18 years by week 108 were not included.

1. Wadhwani S, Mansfield SA, Smith AR, et al. Cardiovascular and thromboembolic events in children and adults with glomerular disease: findings from the Cure GlomeruloNephropathy (CureGN) Network. *Kidney Med*. 2025;7(6):100877. https://doi.org/10.1016/j.xkme.2024.100877

CONSORT 2025 Checklist of Information to Include When Reporting a Randomized Trial

Section / Topic	No	CONSORT 2025 checklist item description	Reported on page no.[a]
**Title and abstract**			
Title and structured abstract	1a	Identification as a randomised trial	Pg 2
	1b	Structured summary of the trial design, methods, results, and conclusions	Pg 2-3
**Open science**			
Trial registration	2	Name of trial registry, identifying number (with URL) and date of registration	Pg 5, 16
Protocol and statistical analysis plan	3	Where the trial protocol and statistical analysis plan can be accessed	Available in the earlier publication of main results from this study, cited on pg 6 of this article
Data sharing	4	Where and how the individual de-identified participant data (including data dictionary), statistical code and any other materials can be accessed	Pg 17
Funding and conflicts of interest	5a	Sources of funding and other support (e.g., supply of drugs), and role of funders in the design, conduct, analysis and reporting of the trial	Pg 16-17
	5b	Financial and other conflicts of interest of the manuscript authors	Pg 16-17
**Introduction**			
Background and rationale	6	Scientific background and rationale	Pg 4-5

Objectives	7	Specific objectives related to benefits and harms	Pg 5
**Methods**			
Patient and public involvement	8	Details of patient or public involvement in the design, conduct and reporting of the trial	N/A
Trial design	9	Description of trial design including type of trial (e.g., parallel group, crossover), allocation ratio, and framework (e.g., superiority, equivalence, non-inferiority, exploratory)	Pg 5
Changes to trial protocol	10	Important changes to the trial after it commenced including any outcomes or analyses that were not prespecified, with reason	Pg 6-7
Trial setting	11	Settings (e.g., community, hospital) and locations (e.g., countries, sites) where the trial was conducted	Available in the earlier publication of main results from this study, as cited on p 5 of this article
Eligibility criteria	12a	Eligibility criteria for participants	Pg 5
	12b	If applicable, eligibility criteria for sites and for individuals delivering the interventions (e.g., surgeons, physiotherapists)	N/A
Intervention and comparator	13	Intervention and comparator with sufficient details to allow replication. If relevant, where additional materials describing the intervention and comparator (e.g., intervention manual) can be accessed	Pg 5
Outcomes	14	Pre-specified primary and secondary outcomes, including the specific measurement variable (e.g., systolic blood pressure), analysis metric (e.g., change from baseline, final value, time to event), method of aggregation (e.g., median, proportion), and time point for each outcome	Pg 6
Harms	15	How harms were defined and assessed (e.g., systematically, non-systematically)	Pg 6-7

Sample size	16a	How sample size was determined, including all assumptions supporting the sample size calculation	Reported in the earlier publication of main results from this study, as cited on p 5 of this article
	16b	Explanation of any interim analyses and stopping guidelines	N/A
Randomisation:			
Sequence generation	17a	Who generated the random allocation sequence and the method used	N/A
	17b	Type of randomisation and details of any restriction (e.g., stratification, blocking and block size)	Pg 5
Allocation concealment mechanism	18	Mechanism used to implement the random allocation sequence (e.g., central computer/telephone; sequentially numbered, opaque, sealed containers), describing any steps to conceal the sequence until interventions were assigned	N/A
Implementation	19	Whether the personnel who enrolled and those who assigned participants to the interventions had access to the random allocation sequence	N/A
Blinding	20a	Who was blinded after assignment to interventions (e.g., participants, care providers, outcome assessors, data analysts)	Pg 5
	20b	If blinded, how blinding was achieved and description of the similarity of interventions	N/A
Statistical methods	21a	Statistical methods used to compare groups for primary and secondary outcomes, including harms	Pg 6-7
	21b	Definition of who is included in each analysis (e.g., all randomised participants), and in which group	Pg 5-6, Fig 1
	21c	How missing data were handled in the analysis	N/A

	21d	Methods for any additional analyses (e.g., subgroup and sensitivity analyses), distinguishing prespecified from post-hoc	Pg 6-7
**Results**			
Participant flow, including flow diagram	22a	For each group, the numbers of participants who were randomly assigned, received intended intervention, and were analysed for the primary outcome	Fig 1
	22b	For each group, losses and exclusions after randomisation, together with reasons	Fig 1
Recruitment	23a	Dates defining the periods of recruitment and follow-up for outcomes of benefits and harms	NR
	23b	If relevant, why the trial ended or was stopped	NA
Intervention and comparator delivery	24a	Intervention and comparator as they were actually administered (e.g., where appropriate, who delivered the intervention/comparator, how participants adhered, whether they were delivered as intended [fidelity])	Pg 5, Fig 1
	24b	Concomitant care received during the trial for each group	N/A
Baseline data	25	A table showing baseline demographic and clinical characteristics for each group	Table 1
Numbers analysed, outcomes and estimation	26	For each primary and secondary outcome, by group:<br>● the number of participants included in the analysis<br>● the number of participants with available data at the outcome time point<br>● result for each group, and the estimated effect size and its precision (such as 95% confidence interval)<br>● for binary outcomes, presentation of both absolute and relative effect size	Pg 8-12, Figs 1-4, Tables 1-2
Harms	27	All harms or unintended events in each group	Pg 11-12, Table 2
Ancillary analyses	28	Any other analyses performed, including subgroup and sensitivity analyses, distinguishing pre-specified from post-hoc	N/A
**Discussion**			

Interpretation	29	Interpretation consistent with results, balancing benefits and harms, and considering other relevant evidence	Pg 12-15
Limitations	30	Trial limitations, addressing sources of potential bias, imprecision, generalisability, and, if relevant, multiplicity of analyses	Pg 14-15

[a]Page numbers refer to the submitted manuscript.

Citation: Hopewell S, Chan AW, Collins GS, Hróbjartsson A, Moher D, Schulz KF, et al. CONSORT 2025 Statement: updated guideline for reporting randomised trials. BMJ. 2025; 388:e081123. https://dx.doi.org/10.1136/bmj-2024-081123.

© 2025 Hopewell et al. This is an Open Access article distributed under the terms of the Creative Commons Attribution License (https://creativecommons.org/licenses/by/4.0/), which permits unrestricted use, distribution, and reproduction in any medium, provided the original work is properly cited.
